# Modeling Inhibitory Interneurons in Efficient Sensory Coding Models

**DOI:** 10.1371/journal.pcbi.1004353

**Published:** 2015-07-14

**Authors:** Mengchen Zhu, Christopher J. Rozell

**Affiliations:** 1 Wallace H. Coulter Department of Biomedical Engineering, Georgia Institute of Technology, Atlanta, Georgia, United States of America; 2 School of Electrical and Computer Engineering, Georgia Institute of Technology, Atlanta, Georgia, United States of America; University of Tübingen and Max Planck Institute for Biologial Cybernetics, GERMANY

## Abstract

There is still much unknown regarding the computational role of inhibitory cells in the sensory cortex. While modeling studies could potentially shed light on the critical role played by inhibition in cortical computation, there is a gap between the simplicity of many models of sensory coding and the biological complexity of the inhibitory subpopulation. In particular, many models do not respect that inhibition must be implemented in a separate subpopulation, with those inhibitory interneurons having a diversity of tuning properties and characteristic E/I cell ratios. In this study we demonstrate a computational framework for implementing inhibition in dynamical systems models that better respects these biophysical observations about inhibitory interneurons. The main approach leverages recent work related to decomposing matrices into low-rank and sparse components via convex optimization, and explicitly exploits the fact that models and input statistics often have low-dimensional structure that can be exploited for efficient implementations. While this approach is applicable to a wide range of sensory coding models (including a family of models based on Bayesian inference in a linear generative model), for concreteness we demonstrate the approach on a network implementing sparse coding. We show that the resulting implementation stays faithful to the original coding goals while using inhibitory interneurons that are much more biophysically plausible.

## Author Summary

Cortical function is a result of coordinated interactions between excitatory and inhibitory neural populations. In previous theoretical models of sensory systems, inhibitory neurons are often ignored or modeled too simplistically to contribute to understanding their role in cortical computation. In biophysical reality, inhibition is implemented with interneurons that have different characteristics from the population of excitatory cells. In this study, we propose a computational approach for including inhibition in theoretical models of neural coding in a way that respects several of these important characteristics, such as the relative number of inhibitory cells and the diversity of their response properties. The main idea is that the significant structure of the sensory world is reflected in very structured models of sensory coding, which can then be exploited in the implementation of the model using modern computational techniques. We demonstrate this approach on one specific model of sensory coding (called “sparse coding”) that has been successful at modeling other aspects of sensory cortex.

## Introduction

The diverse inhibitory interneuron population in cortex has been increasingly recognized as an important component in shaping cortical activity [1]. However, it remains unclear in many settings how the inhibitory circuit specifically contributes to the neural code. While theoretical and simulation investigations of proposed neural coding models could be extremely valuable for providing insight into the role of inhibition, many current high-level functional and mechanistic models do not include inhibitory cell populations that approach the biophysical complexity seen in nature.

Though the main ideas likely extend to other areas, for concreteness we will focus the present discussion on the primary visual cortex (V1). In V1, visual information is encoded using a rich interconnected network of excitatory principal cells and inhibitory cells, and different coding functions appear to be implemented by distinct inhibitory populations [2, 3]. Though V1 has been extensively studied through experiment and modeling, there are often significant discrepancies between what is known about biophysical sources of inhibition and how inhibitory influences are instantiated in a model. For example, in previous high-level functional coding models (e.g. in [4–6], with the exception of [7] as discussed later), neural activity is often treated as a signed quantity without explicitly distinguishing between excitatory and inhibitory cell types. On the other hand, while state-of-the-art large scale mechanistic models (e.g. [8]) typically include a distinct inhibitory population, these types of models often use a single recurrent connectivity pattern (e.g., weights that decrease with spatial separation). This approach results in interneurons with uniform physiological properties and without the complex tuning diversity observed in inhibitory interneurons.

For theoretical and simulation studies to illuminate the role of inhibition in neural coding, it is imperative that coding models begin to incorporate experimental observations regarding the distinct properties of excitatory cells and inhibitory interneurons. Specifically, to realistically investigate the role of inhibition in neural coding, models should incorporate at least three major properties while staying faithful to the coding rule and other desirable properties (e.g., robustness):

1. Inhibitory and excitatory interactions arise from distinct cell types, and synapses from an inhibitory cell cannot have excitatory influences on postsynaptic cells and vice versa (Dale’s law [9]);
2. Excitatory neurons generally outnumber inhibitory interneurons, with E/I ratios recently estimated to be in the range 7:1 to 6:1 (apparently preserved across animals [10, 11]); and
3. The interneuron population has diverse tuning properties [12], including to varying degrees both orientation tuned and untuned interneurons in cat [13] and rodent V1 (reviewed in [14]).

The main contribution of this paper is to demonstrate a systematic computational method for effectively incorporating these biophysical interneuron properties into dynamical systems implementing neural coding models. In our proposed approach we exploit the fact that in many cases of interest, the total required inhibition is highly structured due to the relationship between the coding model and the statistics of the inputs being encoded. Similar to efficient coding hypotheses that postulate compact representations of sensory stimuli, the structure of the sensory statistics and the coding model can also be used to implement the required inhibition with a parsimonious computational structure. Specifically, we propose to reformulate the connectivity matrix to respect Dale’s law and exploit the inhibition structure in a matrix factorization to minimize the number of inhibitory interneurons. Furthermore, we leverage recent results from the applied mathematics community on advanced matrix factorizations to develop an approach that demonstrates the observed diversity of orientation tuning properties in inhibitory interneurons.

The end result of this approach is a network implementation that is functionally equivalent to the original model, but which has an interneuron population that better respects the three major biophysical properties ignored by many current coding models. In addition to this primary goal of providing a recipe for including inhibitory interneurons into coding models, this approach also suggests possible functional interpretations of some biophysical properties of the interneuron population. In particular, we propose that while Dale’s law may reflect a physical constraint of individual cells, in contrast the E/I ratio can be viewed as an emergent characteristic of a population implementation that maximizes efficiency by minimizing the number of interneurons and thus maintenance costs. In addition, we demonstrate that the orientation tuning diversity in the inhibitory population can arise from differential connectivity patterns between the excitatory and inhibitory cells.

## Results

### Network implementation of neural coding models

In a recurrent network implementing a neural coding model, each node in the network is generally driven by both exogenous inputs (i.e., bottom-up inputs due to the stimulus or top-down feedback) and lateral connections from other cells in the same network. These lateral connections are often described in terms of a connectivity matrix *G*, where the element [*G*]_*m*, *n*_ describes synaptic strength from the *n* ^th^ neuron to the *m* ^th^ neuron. While *G* can take many forms, the structure is governed by the coding model and the statistics of the stimuli being encoded.

To illustrate how *G* arises for a family of commonly-used coding models, we consider the Bayesian inference paradigm that has found increasing support as a framework for studying neural coding [15]. While there are many ways to develop a neural coding model based on the ideas of optimal inference, one of the most common approaches is to assume a generative model where the sensory scene is composed of a linear combination of basic features (i.e., causes) that must be inferred. Specifically, a linear generative model for vision proposes that an image patch **s** ∈ ℝ^*N*^ (i.e., an *N*-pixel image patch) can be approximately written as a linear superposition of *M* dictionary elements {*ϕ*_*i*_} representing basic visual features (i.e., there are *M* principal cells):

$$s=\sum_{i=1}^{M}a_{i}\phi_{i}+n=\Phi a+n,$$ (1)

where the coefficients for each feature are {*a*_*i*_}, **n** represents a noise source, and the *N* × *M* matrix Φ consists of one dictionary element on each column. These dictionary elements are often interpreted as the receptive fields (RFs) of a principal cell, such as spiny stellate cells or pyramidal cells.

Given the dictionary Φ and the stimulus **s**, the coefficients **a** in the linear generative model (taken to be principal cell activities, such as instantaneous firing rates) can be found by maximum a posteriori (MAP) estimation. Assuming Gaussian noise and a prior distribution *P*(**a**), the MAP estimate is found by minimizing the negative log of the posterior:

$$E\left(a\right)=\frac{1}{2}{\left\|s-\Phi a\right\|}_{2}^{2}-\lambda \log P\left(a\right),$$ (2)

where *λ* is a scalar capturing the model SNR. When the prior distribution is log-concave (as are many common distributions including the exponential family [16]), the inference can be achieved by simple descent methods. The simplest dynamical system for this coding strategy would be a network implementing gradient descent with population dynamics given by

$$\tau \dot{a}=\Phi^{T}s-Ga+\lambda \nabla \log P\left(a\right),$$

where *τ* is the system time constant and the *M* × *M* recurrent weight (connectivity) matrix is given by *G* = Φ^*T*^Φ. *G* can be interpreted as a recurrent matrix because its off-diagonal terms capture the influence between cell activities. In particular when we assume that the prior is independent, i.e. log *P*(**a**) = ∑_*i*_ log *P*(*a*_*i*_), as is common in efficient coding models, *G* captures *all* the recurrent influence. Note that any dynamical system involving a derivative of an energy function such as Eq (2) will contain a recurrent matrix *G* of this form.

While the most obvious implementation of the network would use a single interneuron for each entry of *G* (connecting two cells), there are many implementations that would result in a functionally equivalent coding rule. For example, one of the approaches we will utilize is to model the connectivity between the interneurons and principal cells using a matrix factorization:

$$G=U\Sigma V^{T}$$

where the *V* ^*T*^ matrix captures the synaptic connections onto a set of interneurons from the principal cells, the *U* matrix captures the synaptic connections from these interneurons back onto the network of principal cells, and Σ is a diagonal matrix representing the independent gains/sensitivity of each interneuron.

### Example: Sparse coding

As a concrete relevant example, we will demonstrate the proposed approach in the context of a dynamical system implementing a sparse coding model of V1, where a population of cells encodes a stimulus at a given time using as few active units as possible. The sparse coding model (combined with unsupervised learning using the statistics of natural images) has been shown to be sufficient to explain the emergence of V1 classical and nonclassical response properties [17–19], potentially has many benefits for sensory systems [20–23], and is consistent with many recent electrophysiology experiments [24–26]. The sparse coding model has been implemented in networks that have varying degrees of biophysical plausibility (e.g., [18, 27–30]), though this model has rarely been implemented with distinct inhibitory neural populations (excepting [7], discussed later).

The sparse coding model can be viewed as a special case of inference in the linear generative model described above with

$$E\left(a\right)=\frac{1}{2}{\left\|s-\Phi a\right\|}_{2}^{2}+\lambda {\left\|a\right\|}_{1},$$ (3)

where $\|\text{a}{\|}_{1}=\sum_{i=1}^{M}|a_{i}|$, corresponding to a Laplacian prior with zero-mean. We base our discussion on a dynamical system proposed in [27] that uses neurally plausible computational primitives to implement sparse coding. This system has strong convergence guarantees [31, 32], can implement many variations of the sparse coding hypothesis [33], and is implementable in neuromorphic architectures [29, 34, 35]. Specifically, the system dynamics for this sparse coding model are:

$$\begin{array}{cc} \dot{u}\left(t\right) & =\frac{1}{\tau }[\Phi^{T}s-u\left(t\right)-\left(G-I\right)a\left(t\right)]\\ a(t) & =T_{\lambda }\left(u\left(t\right)\right), \end{array}$$ (4)

where *I* is the identity matrix, **u** are internal state variables for each node (e.g., membrane potentials), *G* = Φ^*T*^Φ governs the connectivity between nodes, and *T*_*λ*_(⋅) is the soft thresholding function. Note that despite not using steepest descent on Eq (3), this network model still has recurrent connections described by the connectivity matrix *G* = Φ^*T*^Φ. In the simulations in this study, the dictionary Φ is pre-adapted to the statistics of the natural scene with a standard unsupervised learning method, resulting in Gabor wavelet-like kernels that resemble V1 classical receptive fields [17].

**Fig 1:**
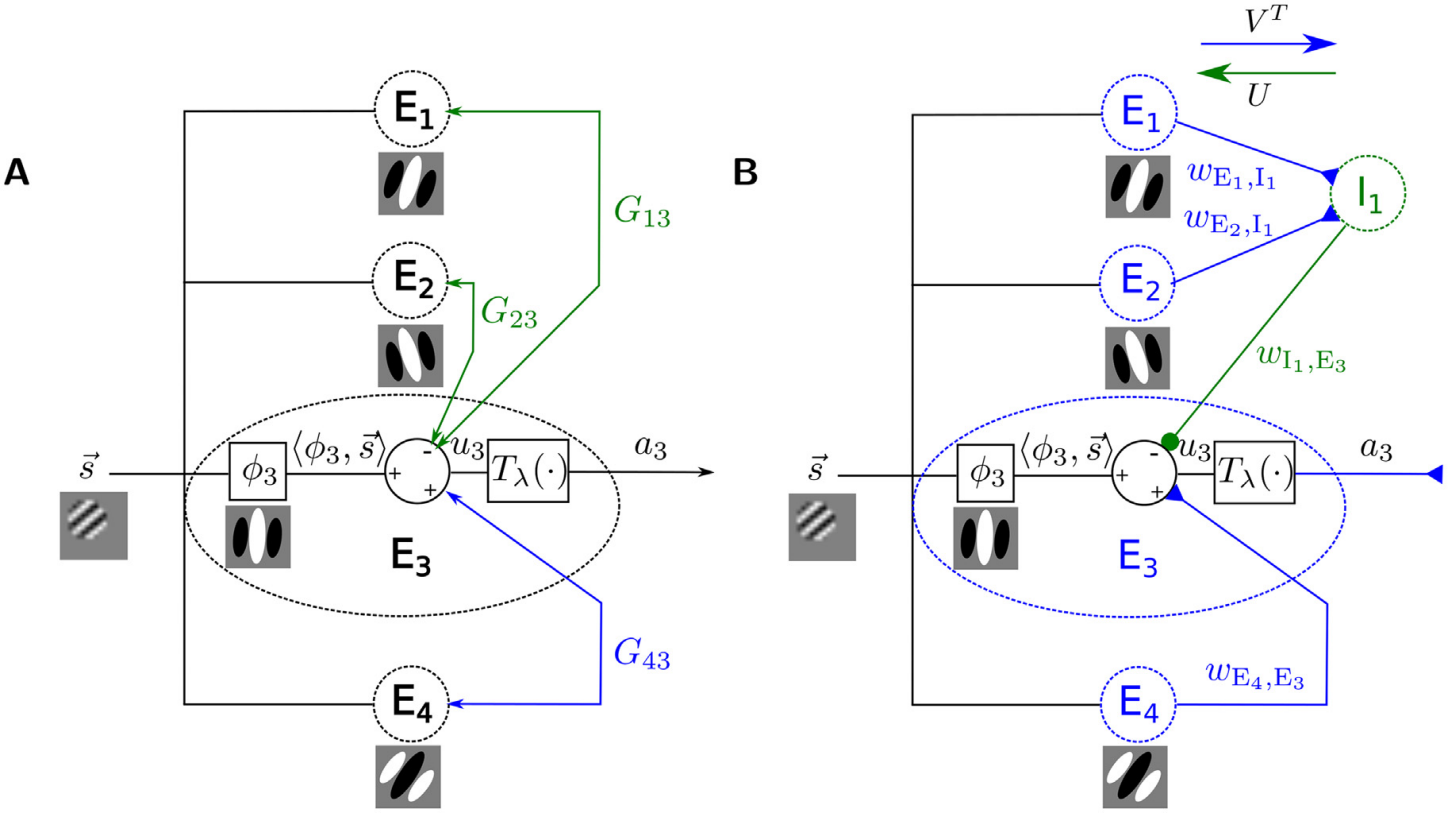
Achieving Dale’s law. (A) An example generic neural network of visual encoding with feedforward and bi-directional recurrent connections (arrows) showing the implementation details of a single cell E_3_ (other cells would be similar but are not pictured for simplicity). The sparse coding dynamics in Eq (4) is a special case. The internal state *u*_3_ (e.g., membrane potential) of this neuron is determined by the filtered input ⟨*ϕ*_3_,**s**⟩, with the dictionary elements *ϕ*’s depending on the natural scene statistics (e.g., [17]), the inhibitory recurrent input (green input *G*_13_ *a*_1_ and *G*_23_ *a*_2_ from E_1_ and E_2_), and the excitatory recurrent input (blue input *G*_43_ *a*_4_ from E_4_). The membrane potential is thresholded by function *T*_*λ*_(⋅) to generate the response *a*_3_ (e.g., the instantaneous spike rate) that drives other neurons. Note that both the excitatory and inhibitory influences are generated by the same generic cell type, violating Dale’s law. (B) In this study, we incorporate distinct inhibitory interneuron populations (e.g. I_1_) that are connected to the principal cells (the E-population) in specific patterns. The computational property of this type of E-I network can be shown to be equivalent to the one in (A).

This dynamical system model requires influences between cells that are described by the matrix *G*, but it is agnostic about the network mechanism that implements these interactions. Specifically, the model as described in [27] does not incorporate a separate population of inhibitory interneurons with any non-trivial interneuron structure, and this naïve description would only imply a point-to-point connection between all pairs of cells in the network as illustrated in Fig 1. This model is therefore unhelpful in its current form for understanding the coding properties of the inhibitory population.

This sparse coding network will serve as a concrete demonstration of the proposed strategy to incorporate more biophysically realistic inhibitory interneurons. The example network we use has 2048 excitatory neurons and has the same parameters as in a previous work [19] (see Materials and Methods).

### Achieving Dale’s law through factorization

As a first step towards a biologically realistic interneuron population encoding model, we show that Dale’s law can be respected in the model by decomposing the recurrent connectivity matrix *G* into matrices representing excitatory and inhibitory interactions. Specifically, the recurrent connectivity matrix *G* can be decomposed into inhibitory and excitatory effects:

$$G=G_{+}+G_{-}=G^{\text{Inhib}}+G^{\text{Excite}},$$ (5)

where *G*_+_ are the positive elements of the matrix (representing the inhibitory recurrent connections) and *G*_−_ are the negative elements (representing excitatory recurrent connections).

While *G* ^Excite^ can be implemented by direct synapses between excitatory principal cells, the inhibitory component *G* ^Inhib^ requires inhibitory interneurons between the relevant principal cells. To capture these disynaptic connections, we factor the inhibitory matrix into two matrices: *G* ^Inhib^ = *UV* ^*T*^. For a simple stylized illustration, the network in Fig 1 shows an example implementation with

$$G^{\text{Inhib}}=\underset{U}{\underset{︸}{\left(\begin{array}{c} 0\\ 0\\ w_{{\text{I}}_{1},{\text{E}}_{3}}\\ 0 \end{array}\right)}}\underset{V^{T}}{\underset{︸}{\;(\begin{array}{cccc} w_{{\text{E}}_{1},{\text{I}}_{1}} & w_{{\text{E}}_{2},{\text{I}}_{1}} & 0 & 0 \end{array})}},$$ (6)

and

$$G^{\text{Excite}}=(\begin{array}{cccc} 0 & 0 & 0 & 0\\ 0 & 0 & 0 & 0\\ 0 & 0 & 0 & 0\\ 0 & 0 & w_{{\text{E}}_{4},{\text{E}}_{3}} & 0 \end{array}).$$ (7)

**Fig 2:**
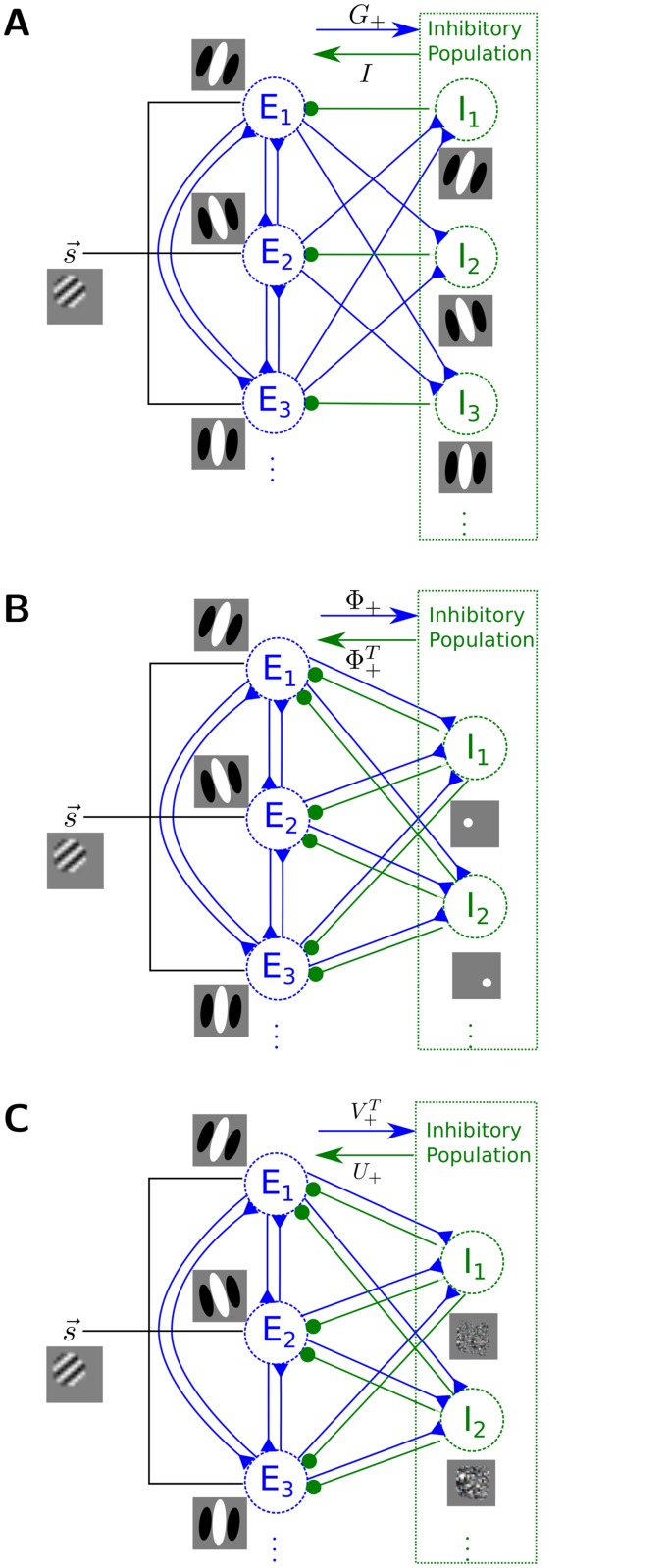
Achieving E/I cell ratio. (A) A subnetwork showing the connectivity and RFs in the network implementation of Eq (8). The excitatory connection weight from E_*i*_ to the inhibitory interneurons I_*j*_ is −⟨*ϕ*_*i*_, *ϕ*_*j*_⟩ (forming the (*i*, *j*)^th^ entry of *G*_+_ in Eq (8)). The recurrent connections from the inhibitory neurons back to the excitatory ones (in green) are one-to-one (rows of the identity matrix). This implementation results in an inhibitory population with similar size and orientation tuning properties as the presynaptic excitatory cells. (B) A stylized sub-network showing the network implementing Eq (9). The RFs (mapped out by sparse dots [17]) of the interneurons are dot-like, with extreme localization and no orientation tuning. (C) A stylized sub-network implementing Eq (11). The interneurons receive excitatory inputs weighted by the corresponding row in $V_{+}^{T}$, adjust the gain by the corresponding diagonal entry in Σ, and projects back to the excitatory population with connectivity weights determined by the corresponding row in *U*_+_. These interneurons receive dense input from many principal cells and have unstructured receptive fields, again with no discernible orientation tuning.

Using the approach above, we can derive a network implementation that is equivalent to the dynamical system instantiating the desired neural coding rule but that also has inhibitory cell properties that can be varied by the choice of factorization for *G* ^Inhib^. For a simple concrete example, we can achieve the same encoding as Eq (4) while incorporating an inhibitory population by using the decomposition:

$$G^{\text{Inhib}}=IG_{+}$$ (8)

where *I* is the identity and plays the role of *U*; *G*_+_ as defined in Eq (5) plays the role of *V* ^*T*^. The resulting network is shown in Fig 2A. While this approach does utilize distinct excitatory and inhibitory sub-populations, it still requires *M* inhibitory neurons (i.e., one for each principal cell) and all inhibitory cells in this implementation have the same orientation tuning properties as the excitatory cells (see Supporting Information S1 Text “RFs of inhibitory cells in the direct implementation”). While this may introduce orientation tuning diversity due to the orientation tunings of the excitatory population, the diversity is distributed uniformly [36] instead of a bimodal dichotomy observed in the inhibitory population [37].

### Achieving E/I ratio through low-rank decomposition

In areas such as V1, the principal excitatory cells are presumed to form the explicit representation of the stimulus that is communicated to higher cortical areas while inhibitory neurons are presumed to play a more localized computational role within a circuit. Using limited physical resources, there are many desirable properties for the stimulus representation: informational efficiency matched to scene statistics [38], stability to small stimulus changes [4], and simple downstream decoding [39]. The principal cell population in V1 appears to be substantially *overcomplete* (i.e., in both cats and primates, the estimated ratio between the output fibers and the input fibers ranges from 25:1 to 50:1 [40]), which is a feature adopted in some coding models because it can help achieve these desirable properties [40].

In contrast, if inhibitory neurons only need to achieve a computational goal for the circuit without requiring these same stimulus coding properties, there is no need for an overcomplete inhibitory population. In fact, the system could exploit this structure to use the fewest number of inhibitory cells possible to avoid incurring unnecessary cell maintenance costs [41]. In contrast to the direct model of Fig 2A, this approach would require interneurons that communicate simultaneously with a *population* of excitatory neurons rather than a single excitatory neuron. As an aside, we note that the reasoning above suggests that the inhibitory population should be overcomplete in systems where these neurons *do* form the explicit stimulus representation. Indeed, this is proposed in a theory of olfactory bulb encoding where granule cell interneurons form the olfactory representation and are an overcomplete population [42].

A natural question to ask is, what is the minimum number of inhibitory cells required to implement the influences specified by the matrix *G*? Said mathematically, what choice of factorization results in the fewest number of inhibitory cells, corresponding to the number of columns of *U* and *V*? In many cases of interest, the connectivity matrix *G* is likely to be low-rank (i.e. *M* > rank(*G*)), providing an opportunity to achieve an efficient implementation of the interneuron population by “compressing” the recurrent connectivity to its most essential components. There are two different causes of low-rank structure in *G* for the types of models considered in this study. First, an overcomplete representation of the principal cells implies directly that *G* is low-rank (i.e., *M* > *N* ≥ rank(Φ) = rank(Φ^*T*^Φ) = rank(*G*)). Second, natural images are highly structured, meaning that image patches have fewer “degrees of freedom” than the number of photoreceptors *N* being used to transduce the image (i.e. *N* > rank(Φ) = rank(*G*)) [43, 44]. This high level of input redundancy means that the connectivity structure implementing this coding rule also has structure that can lead to a simplified implementation. Taking both of these aspects together, models that encode stimuli with low-dimensional structure using an overcomplete code could expect to efficiently implement the encoding rule with highly-structured, low-rank connectivity matrix *G*.

In detail, these two sources of low-rank structure can be exploited to achieve the same coding function of Eq (4) with fewer interneurons than a direct implementation of Eq (8). The original description in Eq (4) of *G* as a Gramian matrix gives rise to the following decomposition of the recurrent matrix:

$$G=\Phi^{T}\Phi ={\left(\Phi_{+}+\Phi_{-}\right)}^{T}\left(\Phi_{+}+\Phi_{-}\right)=\underset{G^{\text{Inhib}}}{\underset{︸}{\Phi_{+}^{T}\Phi_{+}+\Phi_{-}^{T}\Phi_{-}}}+\underset{G^{\text{Excite}}}{\underset{︸}{\Phi_{+}^{T}\Phi_{-}+\Phi_{-}^{T}\Phi_{+}}},$$ (9)

shown in Fig 2B. Assuming first that we only take advantage of an overcomplete representation (i.e. the Φ matrix has more columns than rows because *M* > *N*), the resulting E/I ratio is *M*:*N* and requires (potentially many) fewer inhibitory cells than excitatory cells. However, this implementation does not produce the diversity of tuning properties observed in V1 interneurons, which can be either orientation tuned or non-orientation tuned (with no apparent structure) [37]. In fact, when using sparse dot stimuli to map out the RFs [17] of these interneurons, the resulting RFs have a dot-shaped structure (Fig 2B) inconsistent with cortical observations (see Supporting Information S1 Text “RFs of inhibitory cells in the Gramian decomposition” for discussion relating this RF shape to the network structure).

Further assuming that we exploit the fact that *G* encodes redundant structure in natural scenes, the recurrent connectivity can be represented by an even lower dimensional decomposition than Eq (9). This can be achieved by seeking a lowest-rank (i.e., fewest number of interneurons) recurrent matrix that is also a good approximation to *G* (noting that up to this point we have only examined strategies that *exactly* solve the original encoding problem). Written mathematically, this approximation is:

$$L=\underset{L}{arg min}\text{rank}\left(L\right),\;\text{s.t.}\;{\left\|G-L\right\|}_{F}\leq \epsilon$$ (10)

where ‖⋅‖_*F*_ is the Frobenius norm. This is equivalent to solving:

$$L=\underset{L}{arg min}{\left\|G-L\right\|}_{F},\;\text{s.t.}\;\text{rank}\left(L\right)\leq r$$

with a suitable choice of *r* and *ϵ*. The solution to this problem can be found by the truncated singular value decomposition (SVD), known commonly as Principal Component Analysis (PCA). Note that in our case the truncated singular values are equivalent to the truncated eigenvalues because *G* is symmetric semi-positive definite. Specifically, we can decompose

$$\begin{array}{cc} G & \approx L=U\Sigma V^{T}\\ & =\left(U_{+}+U_{-}\right)\Sigma \left(V_{+}^{T}+V_{-}^{T}\right)\\ & =\left[\underset{G^{\text{Inhib}}}{\underset{︸}{U_{+}\Sigma V_{+}^{T}+\left(-U_{-}\right)\Sigma \left(-V_{-}^{T}\right)}}\right]+\left[\underset{G^{\text{Excite}}}{\underset{︸}{U_{-}\Sigma V_{+}^{T}+U_{+}\Sigma V_{-}^{T}}}\right], \end{array}$$ (11)

where *U* and *V* are truncated singular vectors with orthogonal columns and implement the recurrent synaptic weights (see the Discussion section for the biological plausibility of assuming orthogonal connectivity); Σ is a positive diagonal matrix truncated from the full SVD and implements the interneuron gain (see Materials and Methods).

The resulting inhibitory population receives dense, low-rank connections from the principal cells with synaptic weights defined by $V_{+}^{T}$ (i.e., each row representing synapses convergent onto a single interneuron) as illustrated in Fig 2C. Note that another group of low-rank inhibitory cells with different detailed connectivity is defined by $-V_{-}^{T}$, but the qualitative characteristics of these cells are similar to those defined by $V_{+}^{T}$. Both groups in this population have a gain modulation defined by the diagonals of Σ, followed by projection back to the principal cells with synaptic weights defined by *U*_+_ and −*U*_−_ (i.e., each row represents synapses convergent onto a single principal cell).

In our example sparse coding network, this implementation only requires 220 interneurons to capture about 99% of the variance in *G*, representing a significant savings compared to 2048 and 256 interneurons required in Eqs (8) and (9) respectively. However, the resulting interneurons are again not orientation tuned, lacking the diversity observed in V1 interneurons (Fig 2C). In the Supporting Information S1 Text “RF of inhibitory cells in low-rank decomposition”, we show that the receptive fields of this population approximate the principal components of Φ in a generative linear model and are thus untuned.

### Achieving tuning diversity via convex optimization

Inhibitory neurons are diverse. There are at least two populations with either tuned or untuned orientation selectivity [37]. At the same time, different inhibitory neurons connect to the excitatory population with different frequencies [45]. Could the diverse connectivity contribute to the differences in tuning? It is indeed conceivable that inhibitory neurons densely connected to the excitatory population combine inputs from different sources, and as a result have a broader selectivity. Conversely, inhibitory neurons connecting more sparsely and locally with the excitatory population might be more selective to the stimulus.

To test the hypothesis that tuning diversity could arise from differential connectivity, we decompose the recurrent connectivity matrix into two distinct matrices *L* and *S*

G=L+S, (12)

where *L* is a dense matrix and *S* is a sparse matrix capturing relatively few inhibitory influences in *G*. To also respect the E/I cell ratio constraint, we would like *L* to be low-rank in particular so that a condensed representation could be achieved using SVD as demonstrated in the previous section.

Recently the applied mathematics community has developed a principled algorithmic approach known as Robust PCA (RPCA) [46–48] that effectively solves this decomposition problem. In this approach, a sparse matrix *S* that models “outliers” (having a disproportionate effect on the rank of *G*) is included so that the remainder *L* has a lower rank than *G*.

In the context of our study, an unstructured sparse connectivity matrix can result in a relatively large number of interneurons because there can be a large number of columns containing at least one non-zero value. To maintain the small number of interneurons, we also want the sparse matrix to be row or column-sparse (see for example the connectivity represented in Eq (6)). To achieve this, we used an adaptive version of RPCA (ARPCA) [49] to decompose the recurrent connectivity matrix *G* = Φ^*T*^Φ into a low-rank matrix *L* and a column-sparse matrix *S* by solving the following convex optimization program iteratively:

$$L,S=\arg\min_{L,S}{\left\|L\right\|}_{*}+{\left\|\Lambda S\right\|}_{1},\;\text{subject}\;\text{to}\;G=L+S,$$ (13)

where ‖⋅‖_*_ is the nuclear norm (i.e., the sum of absolute values of eigenvalues) to encourage *L* to have low rank, ‖⋅‖_1_ is the ℓ_1_-norm (i.e., the sum of absolute values of the vectorized matrix) to encourage sparsity, and Λ is a diagonal weighting matrix updated at each iteration to encourage column sparsity in *S*. The update rule for Λ is given by

$$\Lambda_{i,i}=\frac{\beta }{\|S^{\left(i\right)}{\|}_{1}+\gamma },$$ (14)

where *S* ^(*i*)^ is the *i* ^th^ column of *S*, and *β* and *γ* control the speed of adaptation. At each iteration, the columns of *S* with smaller entries are assigned larger values of *λ*, thus encouraging the values in that column to become even smaller and eventually approach zero. The end effect is that the algorithm converges to a decomposition where only a few columns in *S* are non-zero (see Materials and Methods for details). We note that the RPCA formulation in Eq (13) is a natural extension to SVD in Eq (10): instead of constraining the power in *G*−*L* (via the Frobenius norm), we now model this difference using a structured matrix *S*.

After convergence, as before we perform a singular value decomposition (SVD) on the low-rank matrix *L* = *U*Σ*V* ^*T*^. To respect Dale’s law we separate out the excitatory and inhibitory influence similar to Eq (9) in each matrix:

$$\begin{array}{cc} G & =L+S=U\Sigma V^{T}+S\\ & =\left(U_{+}+U_{-}\right)\Sigma \left(V_{+}^{T}+V_{-}^{T}\right)+\left(S_{+}+S_{-}\right)\\ & =\left[\underset{G^{\text{Inhib}}}{\underset{︸}{U_{+}\Sigma V_{+}^{T}+\left(-U_{-}\right)\Sigma \left(-V_{-}^{T}\right)+S_{+}}}\right]+\left[\underset{G^{\text{Excite}}}{\underset{︸}{U_{-}\Sigma V_{+}^{T}+U_{+}\Sigma V_{-}^{T}+S_{-}}}\right]. \end{array}$$ (15)

**Fig 3:**
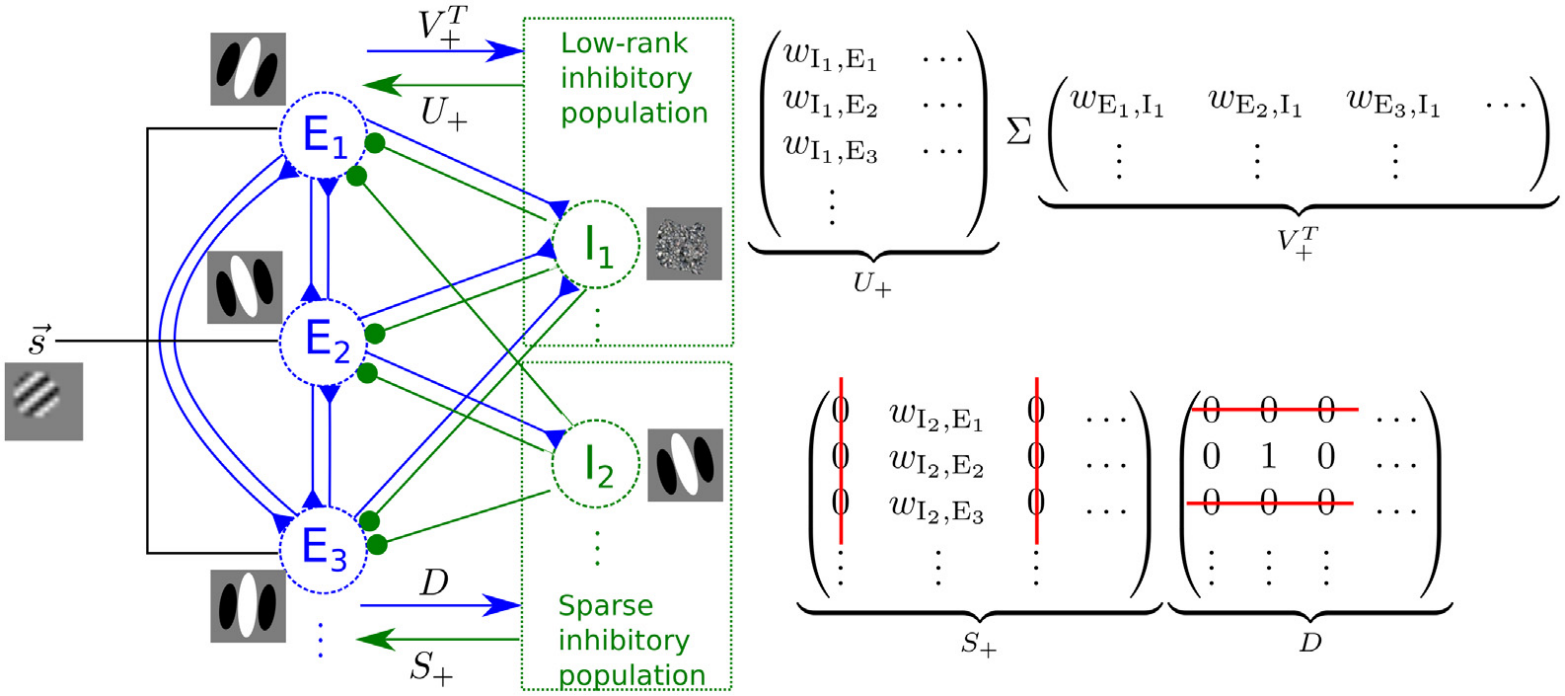
Low-rank plus sparse decomposition of the recurrent connectivity matrix. On the left we show a stylized network structure of the model with low-rank plus sparse decomposition of the recurrent connectivity matrix. The first inhibitory neuron I_1_ belongs to the low-rank subpopulation. The second inhibitory neuron I_2_ belongs to the sparse subpopulation. It receives inputs from a single excitatory neuron (E_2_ in this illustration) with the connectivity matrix implemented by the diagonal matrix *D*, and sends projections back to the excitatory population with weights determined by a non-zero column of the connectivity matrix *S*_+_. This inhibitory cell has the same receptive field as E_2_. The matrices on the right show the decomposition of the recurrent inhibitory connections exemplified in the network on the left. The low rank and sparse inhibitory populations together implement the recurrent inhibition −*G* ^Inhib^. The excitatory recurrent influences are implemented by direct connections *I*−*G* ^Excite^ between the principal cells.

With this decomposition, the recurrent matrix can be rewritten with separate excitatory and inhibitory recurrent interactions. In the sparse coding model example described earlier (Eq (4)), the equivalent network dynamics are:

$$\dot{u}\left(t\right)=\frac{1}{\tau }\left[\underset{\text{feed-forward}}{\underset{︸}{\Phi^{T}s}}-\underset{\text{recurrent inhibitory}}{\underset{︸}{\left(\underset{\text{low-rank}}{\underset{︸}{U_{+}\Sigma V_{+}^{T}+\left(-U_{-}\right)\Sigma \left(-V_{-}^{T}\right)}}+\underset{\text{sparse}}{\underset{︸}{S_{+}D}}\right)}}a\left(t\right)+\underset{\text{recurrent excitatory}}{\underset{︸}{\left(I-G^{\text{Excite}}\right)}}a\left(t\right)-u\left(t\right)\right],$$ (16)

where *D* is a diagonal matrix with 0s and 1s on the diagonal and represents the synaptic weights on the sparsely-connected interneurons made by the principal cells (Fig 3).

With a parameter choice that strikes a balance between sparseness and low rank (see Materials and Methods), the E/I cell ratio in the model network is also close to the observed ratio. Specifically, with 2048 principal cells and 320 inhibitory interneurons (220 in the low rank population and 100 in the sparse population), the model network has an E/I cell ratio of 6.4:1.

Decomposing the connectivity matrix in this manner results in two distinct populations of inhibitory interneurons with a relative size controlled by the magnitude of the average weights in the matrix Λ. The first subpopulation (exemplified by the inhibitory cell I_1_ in Fig 3) originates from the low-rank connectivity matrix *L*, and has properties described in the previous section. The second subpopulation (exemplified by I_2_ in Fig 3) originates from the sparse connectivity matrix *S*. This population receives one-to-one (i.e. sparse) connections with unit weights defined by the diagonal matrix *D* from the principal cells, and projects back to the principal cells with weights defined by *S*. Because *S* is column-sparse, the rows in *D* that correspond to the zero columns in *S* can be set to 0 without altering the recurrent influence. Said another way, we can eliminate the zero rows of the *D* matrix and the zero columns of *S*, meaning that only a few interneurons are required in this subpopulation (Fig 3).

**Fig 4:**
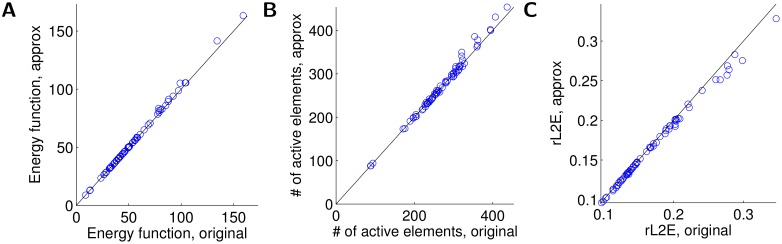
The network implements efficient coding. Comparison of original sparse coding network model to approximation with idealized interneurons. Different markers represent results using different stimuli. (A) The energy function representing the total objective being optimized. (B) The sparsity of the response **a**. (C) The relative ℓ^2^ error of the image reconstruction.

This model network accurately solves the sparse coding inference problem (Eq (3)), despite using only the top principal components of *L* in the approximation to the recurrent matrix. This is shown in Fig 4, where we compare the original network (i.e., the idealized implementation of Eq (4) that is not biophysically plausible) with the approximation described above in the metrics of interest. Specifically, for a number of grating test patches we plot the final value of the energy function (i.e., the quantity to be minimized in Eq (3)), along with the individual quantities relevant to the objective: the sparsity of the final answer (measured by the number of active coefficients ‖**a**‖_0_) and the relatve ℓ^2^ error for the input image (‖**s**−Φ**a**‖_2_/‖**s**‖_2_). As demonstrated in Fig 4, the approximation achieves performance very similar to the original (mean relative error of energy approximation 0.008±0.001). We note specifically that in both the approximated and the original network, the activity is very sparse—only up to 5% of all 2048 neurons are active.

**Fig 5:**
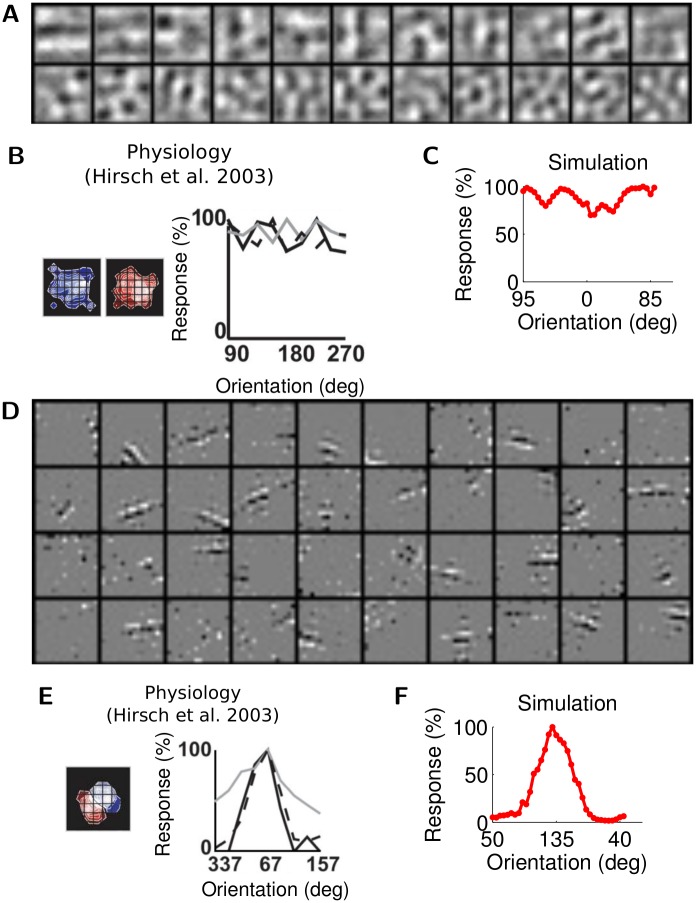
Achieving tuning diversity. (A) Example RFs of the low-rank subnetwork of inhibitory interneurons in the simulation. (B) An example RF and orientation tuning curve from physiological recordings (Reprinted by permission from Macmillan Publishers Ltd: Nature Neuroscience, Fig 7c from [37]); (C) An example orientation tuning curve from the simulation. (D) Example RFs of the sparse subnetwork of inhibitory interneurons. (E) An example RF and orientation tuning curve from physiological recordings (Reprinted by permission from Macmillan Publishers Ltd: Nature Neuroscience, Fig 4d from [37]); (F) An example orientation tuning curve from the simulation.

Interestingly, the sparse and low-rank interneuron populations in RPCA show the same kind of diverse orientation tuning as V1 inhibitory cells in vivo. The low-rank inhibitory population has RFs that are mostly untuned (Fig 5A and 5C; orientation tuning mapped using a grating stimulus centered in the middle of the visual field), comparable to the untuned inhibitory neurons observed in cats [37] (Fig 5B). The sparse inhibitory population has RFs that resemble the primary cell RFs in Φ and are orientation tuned (Fig 5D and 5F; orientation tuning mapped using a grating stimulus centered on the RF of the interneuron), comparable to the tuned inhibitory neurons observed in cats [37] (Fig 5E). This tuning dichotomy is expected from the difference in connectivity: the orientation-tuned inhibitory RFs arise from orientation-selective inputs from single principal cells (i.e., sparse synaptic connections), whereas untuned RFs arise from dense synaptic inputs from many principal cells of different tunings.

**Fig 6:**
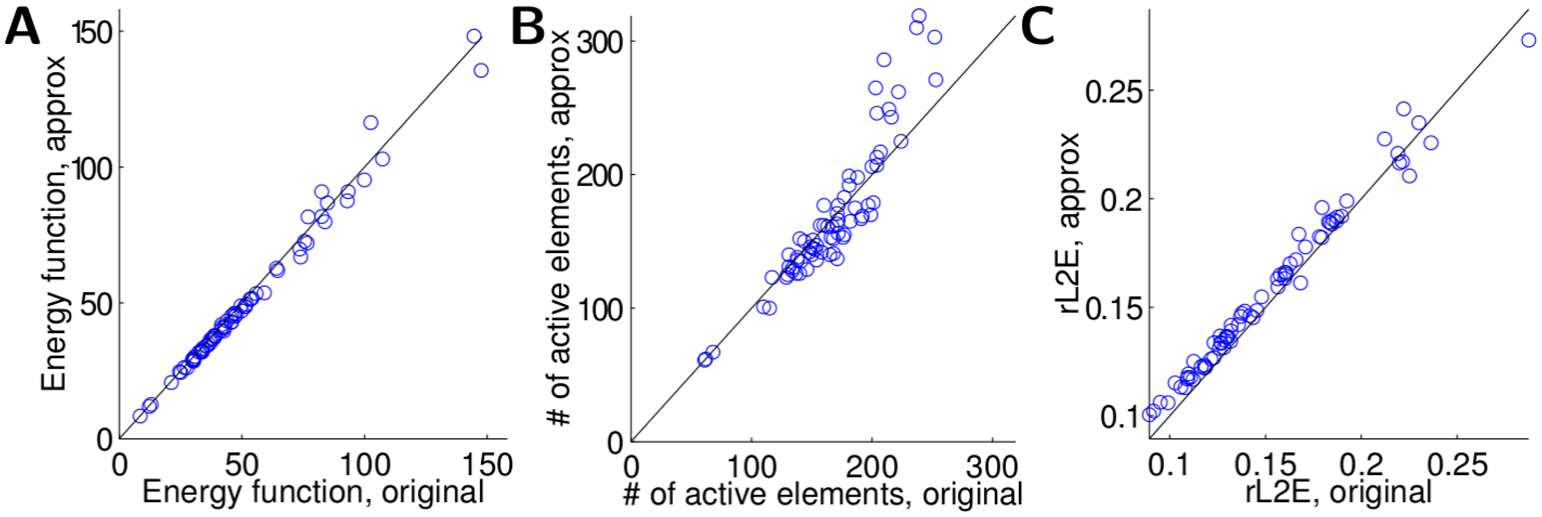
A network with dynamic interneurons implements sparse coding. Comparison of original sparse coding network model to approximation with plausible interneurons with a dynamical model. Different markers represent results using different stimuli. (A) The energy function representing the total objective being optimized. (B) The sparsity of the response **a**. (C) The relative ℓ^2^ error of the image reconstruction.

For simplicity in the above model we treat the interneurons as instantaneous linear units (Eq (16)). To make the model more biologically realistic, we can incorporate the same first-order dynamics (i.e., leaky integration) used by the principal cells into the interneurons. Specifically, the full population dynamics can be written as:

$$\begin{array}{cc} \dot{u}\left(t\right) & =\frac{1}{\tau }[\Phi^{T}s-(U_{+}a_{\text{I,}\;\text{L1}}\left(t\right)+\left(-U_{-}\right)a_{\text{I,}\;\text{L2}}\left(t\right)+S_{+}a_{\text{I,}\;\text{S}}\left(t\right))+(I-G^{\text{Excite}})a\left(t\right)-u\left(t\right)]\\ a(t) & =T_{\lambda }\left(u\left(t\right)\right)\\ {\dot{a}}_{\text{I,}\;\text{L1}}\left(t\right) & =\frac{1}{\tau }\left(\Sigma V_{+}^{T}a\left(t\right)-a_{\text{I,}\;\text{L1}}\left(t\right)\right)\\ {\dot{a}}_{\text{I,}\;\text{L2}}\left(t\right) & =\frac{1}{\tau }\left(\Sigma \left(-V_{-}^{T}\right)a\left(t\right)-a_{\text{I,}\;\text{L2}}\left(t\right)\right)\\ {\dot{a}}_{\text{I,}\;\text{S}}\left(t\right) & =\frac{1}{\tau }\left(Da\left(t\right)-a_{\text{I,}\;\text{S}}\left(t\right)\right), \end{array}$$ (17)

where **a**_I, L1_(*t*) and **a**_I, L2_(*t*) are the dynamic responses of the two low rank interneuron populations and **a**_I, S_(*t*) is the response of the sparse population. Here we assume that inhibitory neurons have the same time constant as the principal cells. As shown in Fig 6, the model defined in Eq (17) still accurately solves the original sparse coding problem (mean relative error of energy approximation 0.029±0.004). Note that due to the added dynamics, the new dynamical system needs more numerical integration steps to converge (all systems run for 100 steps in Fig 6 vs. 25 steps in Fig 4, resulting in some differences in the sparsity-rMSE tradeoff).

## Discussion

The main contribution of this study is a biologically plausible computational framework for including inhibitory interneurons in efficient dynamical system models of neural coding based on ideas from matrix factorization and convex optimization. From the demonstrated results, we conclude that techniques such as low-rank plus sparse decomposition can be used to find implementations of a recurrent connectivity matrix that produce equivalent population dynamics while using an inhibitory structure that matches many biophysical properties, including respecting Dale’s law, known E/I cell ratios, and diversity of orientation tuning properties. In our example of a network implementing sparse coding, the resulting representation is nearly as accurate as the idealized coding model while being much more faithful to the biophysics of the inhibitory population. Because the proposed approach only depends on the structure of the recurrent matrix (which may be common among many energy based models, including many other derivatives of sparse coding [33]), we expect that the results will be applicable to many dynamical systems implementing neural coding models.

Our approach suggests that the excitatory to inhibitory cell ratio in V1 is an emergent property of interneurons implementing efficient visual coding in a resource-conserving way. Specifically, in our model a comparatively small number of interneurons efficiently route the inhibitory influence by taking advantage of the overcomplete and low-rank (redundant) structure in the recurrent connectivity pattern. We have further demonstrated that the tuning diversity of interneurons could arise from differential connectivity with the excitatory population—a prediction that could be tested experimentally.

### Related studies

Recently a few studies explicitly introduced inhibitory interneuron populations into high-level functional encoding models. Lochmann et al. [50] developed a generative model that demonstrates contextual effects in sensory coding and includes a population of inhibitory neurons. These inhibitory cells contribute to efficient perceptual inference through input targeted divisive inhibition. However, this model only works with binary one dimensional inputs and the inhibitory connectivity pattern predicted by this model presently lacks anatomical support at the cortical level. Therefore, its connection with realistic visual encoding remains unclear.

In a more recent work, Boerlin et al. [51] illustrated a way to include a separate inhibitory population in an efficient coding spiking network that estimates the state of an arbitrary linear dynamical system. While providing a spiking model for the inhibitory cells, their approach did not investigate the issues of excitatory-inhibitory cell ratio and tuning diversity. It should also be noted that the Gram recurrent matrix in our model also occurs in their model (their Eq 10). It is therefore possible that our approach could be applied in their scenario.

Another recent study [7] has developed a spiking sparse coding network based on [28] that incorporates a population of inhibitory cells with connectivity weights adapted to natural scenes. Similar to the results of our study, the work in [7] has found that a relatively small number of inhibitory cells are sufficient to provide recurrent competition required for sparse coding. In contrast, the present study formulates a framework for including biologically plausible inhibitory interneurons in a wide range of models in a way that can potentially be proven equivalent computationally to the original model objective (e.g., Eq (2)). Furthermore, the present work captures the observed tuning diversity of inhibitory interneurons in V1. We note that the work in [7] does use a more biophysically realistic learning rule, whereas the present paper uses a global convex optimization approach on a fixed connectivity matrix that may have been established through a learning process.

### Model predictions on the interneuron properties

Our model gives several experimentally verifiable predictions of interneuron properties that we detail in this section. We also note that while biologically plausible, there are limitations with the current model (see the Caveats section later).

First of all, our model predicts the existence of two distinct connectivity patterns between inhibitory interneurons and principal cells: the recurrent connections between principal cells and the low-rank interneurons are dense while the recurrent connections between the principal cells and the sparse interneurons are selective. According to these patterns, a likely biological correlate for the low-rank interneurons in mice is the fast-spiking parvalbumin-expressing (PV) interneurons, which receive dense synaptic inputs from nearby pyramidal cells of diverse selectivities [52], and project densely back to neighboring pyramidal cells [53]. Interestingly, as predicted by our model, the PV neurons indeed have broader selectivity than principal cells [54]. Similarly in cats, a subgroup of fast-spiking interneurons were found to have broader tunings than other interneurons [55]. Note that this broad selectivity means that the interneuron population derived from the low-rank component will use a dense code (i.e., most cells participating for most stimuli) even in coding rules such as the sparse coding example used in this work.

It is less clear what biological correspondence is most appropriate for the sparse interneuron population arising in the model. One candidate is the irregular firing cannabinoid receptor-expressing (CB1+) neurons, which have been shown to be more sparsely connected to the principal cells than the PV neurons [45]. However it is unclear what selectivity properties these neurons have in the visual cortex. Another candidate is the somatostatin expressing (SOM) neurons, which are orientation selective and have weaker response [54], similar to the sparse population in our model. If they indeed correspond to the sparse population in our model, we predict that these neurons receive sparser connections from the principal cells compared to the PV neurons (this however might differ from layer to layer, as evidenced by a recent study in L2/3 [56]).

**Fig 7:**
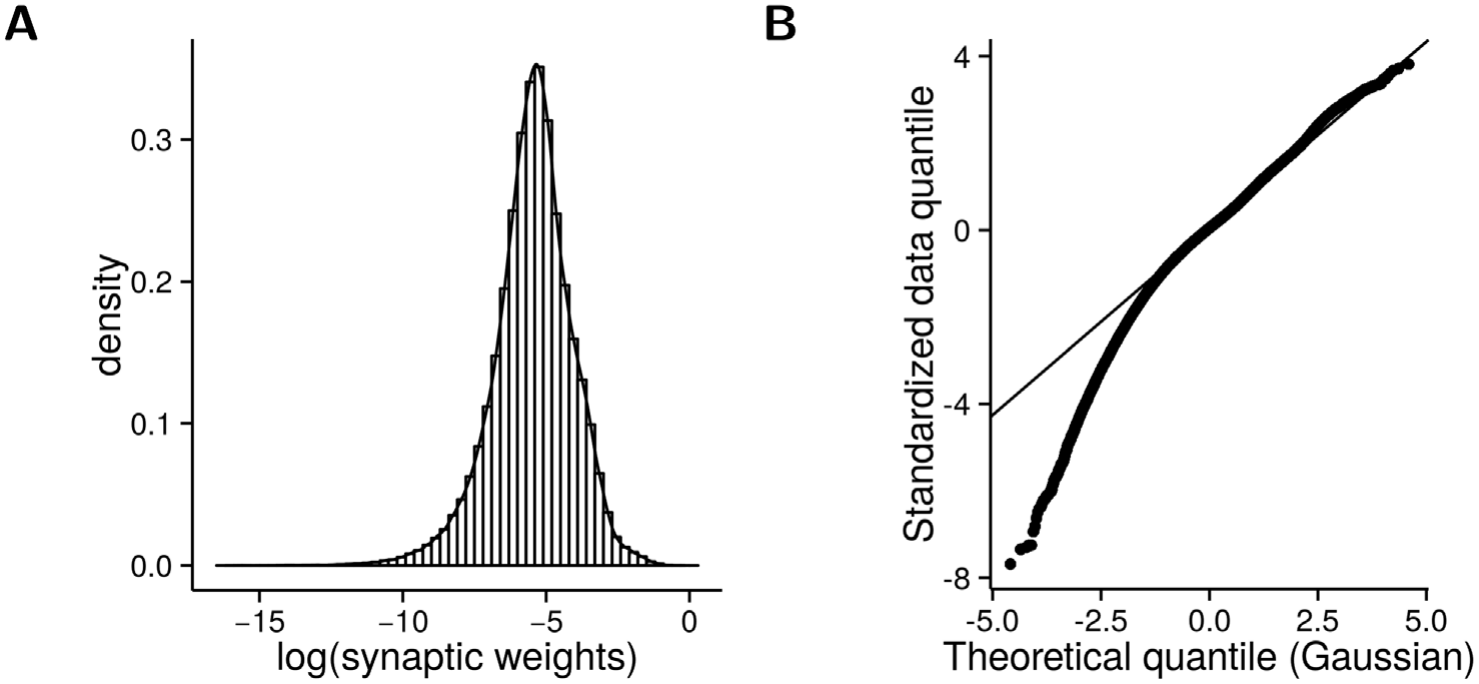
Distribution of synaptic weights. (A) The non-zero inhibitory synaptic weights in the RPCA model have a near log-normal distribution. (B) The Quantile-Quantile (QQ) plot of the starndardized log of the model distribution vs. a standard normal distribution. A line is drawn through the 25% and 75% quantile to illustrate the goodness of fit. The model distribution has a visible tail towards the smaller weights.

In addition to general connectivity patterns, our model also provides predictions on the distribution of inhibitory synaptic weights in V1. As shown in Fig 7A, we observe a near log-normal distribution of the inhibitory synaptic weights when using a dictionary adapted to the statistics of natural scenes. Compared to a standard log-normal distribution however, the model distribution has a longer tail towards the smaller values as visible from the Q-Q plot (Fig 7B). Note that while the heavy tail is significant, in fact only a small part of the distribution deviates from log-normal (below the -2.33 quantile—corresponding to 1% of the cumulative density). Compounded with the difficulty of measuring from weak synapses, we anticipate that this tail would be hard to capture from experimental measurements. We note that there was a previous study [57] demonstrating a log-normal distribution between excitatory neurons, but we are unaware of similar findings for inhibitory cells. It should be noted that this model distribution is in agreement with the prediction of a previous model of spiking sparse coding [28]. Whether this is true in physiology requires further experimental validation.

In discussing the recurrent connections in the network of Fig 3, we concentrate mostly on the inhibitory connections represented by the *G* ^Inhib^ term. The excitatory influences are assumed to be implemented by direct excitatory-excitatory connections represented by the connectivity matrix *I*−*G* ^Excite^. The identity matrix *I* is assumed to be implemented by an independent mechanism that results in self-excitation. Biologically, there are at least three ways this self-excitation could be achieved: through “autapses” [58] (although most of these self-connections were observed in inhibitory cells); through excitatory interneurons that connect back to the principal cells; or through dendritic back-propagation [59].

### Caveats

We note that some of the biological features of inhibitory circuits modeled in this work are still controversial among physiology studies. For example, although Dale’s law is a generally accepted operating principle, it was recently suggested that neurons can segregate neural transmitters to different synapses [60]. As another example, the diversity of tuning properties and the functional roles of inhibitory interneurons are still controversial. Most studies on this topic were conducted in rodents (the study we compared our simulation to [37] in the Results being a notable exception), with few implications for primates and leaving substantial uncertainty even in mouse neocortex [14]. For example, it is still unclear whether PV interneurons have a diversity of tuning properties [61] or are mostly broadly tuned [62]. In addition, in our simulation the recurrent inhibition sharpens the orientation tuning of the principal cells [19]; in physiology, there are conflicting accounts of whether this is the case [2, 3]. In summary, the modeling results here should be considered as a demonstration of the capability of a theoretical model to reproduce a variety of detailed biological phenomenon, not as support for any specific anatomical inhibitory circuit structures and functions.

There are several biological details of the inhibitory population that the current model does not capture. First, the non orientation-tuned inhibitory interneurons in cat primary visual cortex have complex cell characteristics such as overlapping ON/OFF receptive fields (Fig 5B). To capture such features, a coding model involving complex cells may be necessary. Second, the current model does not attempt to capture the prevalent electrical and chemical interconnections between inhibitory interneurons in the cortex [1, 63]. These recurrent connections can potentially be incorporated by allowing off-diagonal entries in the gain matrix Σ. Third, we have treated inhibitory interneurons as continuous-time units with instantaneous dynamics (Eq (16)) or with first-order dynamics (Eq (17)). In reality, interneurons emit spikes and have diverse temporal dynamics involving short-term plasticity [64]. A previous work from our group [35] showed that the non-spiking sparse coding network (without a separate inhibitory population) can be equivalently implemented by a network of integrate and fire cells. While we would expect a spiking network with a similar connectivity pattern as we have demonstrated would exhibit similar kind of interneuron properties, it is unclear without further analysis whether using more biologically realistic spiking neurons would affect the overall dynamics. Finally, though it is well-known that thalamic inputs innervate inhibitory interneurons constituting feedforward inhibition [1], the model discussed in the main text does not include a detailed model of this feedforward component. However, we argue in the Supporting Information S1 Text “Feedforward inhibition” section that the cell ratio and orientation tuning properties could be modeled in a similar manner as the recurrent network.

It is known that neural network models with different parameters may share the same input-output functionality [65]. Similarly, there are other model configurations (i.e. inhibitory connection patterns) not considered in this work that could implement the same coding functionality. For one example, in the Supporting Information S1 Text “Global inhibition” section we consider the example of global inhibition structures. In this case, while very few inhibitory cells are needed, only non orientation-tuned inhibitory cells can be modeled.

A remaining question is whether the proposed decomposition can be learned in a biologically plausible way. While it is out of the scope of the current study, we do expect the orthonormal low-rank connectivity matrices to be learnable in a biologically plausible fashion. Indeed, with Sanger’s learning rule—a classical unsupervised learning method for feedforward neural networks that can be implemented locally—the network weights converge to orthonormal eigenvectors of the input [66]. Note that while the orthonormality emerges automatically from the learning rule, we are not suggesting that the singular vectors are the only plausible weights in the interneuron network. For example, performing a linear transform (e.g. a rotation) in the low-rank principal subspace gives rise to an alternative decomposition that maintains the cell ratio and tuning properties we have modeled. This alternative implementation may in fact have additional computationally benefits. For example, a linear transform equalizes the gain distribution in the SVD and potentially improves the robustness of the network against noise.

## Materials and Methods

### Adaptive Robust PCA

Eq (13) is a convex optimization problem that can be solved efficiently through numerical optimization techniques. In this study we solve this optimization problem through an adaptive version of Alternating Direction Method of Multipliers (ADMM), a robust dual ascent method [67]. Specifically, the inner loop of the algorithm finds the optimal *L* and *S* given a choice of Λ by alternating between a primal update that achieves (augmented) Lagrangian minimization and a dual update. The outer loop updates Λ according to Eq (14). See [49] for details of the algorithm.

### Implementation details

We start with a model network of 2048 principal neurons with receptive fields adapted to 16 × 16 natural image patches using sparse coding [17]. The principal cell activities are interpreted as the sparse coefficients of a dynamical system implementing sparse coding (**a** in Eq (4) constrained to be positive) [27] with a threshold value *λ* = 0.1, as was done previously in [19].

In the proposed implementation, the required number of inhibitory interneurons is governed by the rank of *L* and the number of non-zero columns in *S*. To achieve a biophysically accurate small E/I cell ratio, we would like both the rank of *L* and the number of non-zero columns of *S* to be small. However, these are two competing requirements whose tradeoff depends on the parameters in Eqs (13) and (14). Indeed, making *L* lower rank necessarily makes *S* less column-sparse. To find a compromise solution, we chose the following set of parameters: the initial diagonal of Λ is 0.038; *α* = 2.5; *β* = 0.01. After convergence, we chose to keep 110 cells (implementing top 110 eigenvalues in *L*) in each of the two low-rank inhibitory populations with a total of 220 cells so that 99% of the variance in *L* was retained. We also used 220 interneurons in the SVD implementation to facilitate comparison between the models.

## Supporting Information

S1 Text Mathematical derivations of model receptive fields; models of feedforward inhibition and global inhibition. (PDF)

S1 Fig Feed-forward inhibition. Feedforward push-pull could also be implemented with fewer inhibitory neurons than excitatory neurons.
(TIF)

S2 Fig Global inhibition. (**A**) The recurrent network that implements the global inhibition (Eq. (S8)). I_1_ pools all activities from the excitatory population, weighs them by *c*, and projects back to the excitatory population. (**B**) The orientation tuning curve of the inhibitory neuron I_1_.
(TIF)
