# Supplementary material for: Modeling Inhibitory Interneurons in Efficient Sensory Coding Models
Source: PLoS Comput Biol. 2015 Jul 14;11(7):e1004353. doi: 10.1371/journal.pcbi.1004353 (PMC4501572; doi:10.1371/journal.pcbi.1004353)
Supplement: S1 Text — (PDF) [file pcbi.1004353.s001.pdf]

# Supporting Information: Text S1

Mengchen Zhu[1], Christopher J. Rozell[2,*]
**1 Mengchen Zhu: Wallace H. Coulter Department of Biomedical Engineering, Georgia Institute of Technology, Atlanta, GA 30332 USA;**
**2 Christopher J. Rozell: School of Electrical and Computer Engineering, Georgia Institute of Technology, Atlanta, GA 30332 USA.**
∗ **E-mail: crozell@gatech.edu**

# Mathematical derivations of model receptive fields

To study the orientation tuning of the model cells, we map the receptive field using sparse spots of light as in [1]. In the following derivation, without loss of generality we consider variables that could be both positive and negative instead of constraining the signs as in the main text.

## RFs of excitatory cells

Here we derive the RFs of principal cells in a linear generative model. The stimuli array of sparse dots used for mapping RF is represented by an identity matrix $I = [\mathbf{s}_1, ..., \mathbf{s}_M]$, and the rows of the response matrix $A$ are the RFs.[1] In a linear generative model:

$$I \approx \Phi A. \tag{S1}$$

First consider the case when $\Phi$ (and thus $\Phi^T\Phi$) is full rank. The receptive field mapped with sparse dots is the Moore-Penrose inverse of the dictionary matrix:

$$A \approx \Phi^\dagger = (\Phi^T\Phi)^{-1}\Phi^T. \tag{S2}$$

Specifically when $\Phi$ is orthogonal,

$$A \approx \Phi^T, \tag{S3}$$

indicating that the RFs of the excitatory cells are approximately the same as the dictionary elements.

For an arbitrary overcomplete $\Phi$, there is no unique solution to Eq. (S1). If one regularizes the problem with a sparsity constraint on the columns of $A$, $A$ can be inferred using a linear dynamical system coupled with a nonlinearity (Eq. (4)) [2]. The solution at steady state under sparse dots stimuli, ignoring the thresholding ($\mathbf{a} \approx \mathbf{u}$) can be written as:

$$A \approx \Phi^T I - (\Phi^T\Phi - I)A, \tag{S4}$$

which implies

$$\Phi^T\Phi A \approx \Phi^T, \tag{S5}$$

similar to Eq. (S2). When the dictionary elements are sufficiently different from one another (i.e. $\Phi^T\Phi$ is close to identity), again we have Eq. (S3). Empirically in simulations we observe that the tuning property of the RFs are indeed similar to that of the dictionary when using an overcomplete dictionary learned from natural scenes. A similar observation was also made previously in [1].

[1] Here we mix the signs in the representation for the sake of brevity. In simulations we separate out the signs: we map the ON part of the RF with positive-valued dot, and the OFF part by the negative-valued.

### RFs of inhibitory cells in the direct implementation

When the Gramian matrix is decomposed directly (Eq. (8)), the RFs of the inhibitory cells are $(\Phi^T\Phi)_+A$. According to Eq. (S5), this is approximately the same as the dictionary elements (orientation tuned).

### RFs of inhibitory cells in the Gramian decomposition

When the Gramian matrix $\Phi^T\Phi$ is used to represent the recurrent inhibition directly (Fig. 2**B**), the RFs of the interneurons are columns of $\Phi A$, where columns of $A$ are the responses of principal cells to sparse dots stimuli. It is straightforward to infer from Eq. (S1) that the RFs of the interneurons are dots (simulation in Fig. 2**B**) since $\Phi A$ is the identity matrix.

### RFs of inhibitory cells in low-rank decomposition

Denote the SVD of $\Phi$ as

$$\Phi = \Omega\Lambda\Upsilon^T, \tag{S6}$$

so that

$$\Phi^T\Phi = \Upsilon\Lambda\Omega^T\Omega\Lambda\Upsilon^T = \Upsilon\Lambda^2\Upsilon^T. \tag{S7}$$

From Eq. (13), since $S$ is sparse, $\Phi^T\Phi \approx L = U\Sigma V^T$. Therefore $U \approx \Upsilon \approx V$ and $\Sigma \approx \Lambda^2$ up to scaling constants. The RFs of the inhibitory neurons implementing the low-rank part of the connectivity matrix can thus be approximated up to a scaling constant by:

$$V^TA \approx \Upsilon^TA = (\Lambda^{-1}\Omega^T\Omega\Lambda)\Upsilon^TA = (\Omega\Lambda^{-1})^T(\Omega\Lambda\Upsilon^TA) = (\Omega\Lambda^{-1})^T(\Phi A) \overset{(S1)}{\approx} (\Omega\Lambda^{-1})^T.$$

In words, this means that the RFs of these interneurons can be approximated by the (weighted) singular vectors of the dictionary $\Phi$. Since the dictionary is learned from natural scenes, the singular vectors resemble the PCA components learned from natural scenes, which are known to be sinusoidal [3]. The simulation confirms this (Fig. 5**A**).

## Feedforward inhibition

While the main text addressed the role of inhibition in the recurrent cortical connections, similar considerations arise when modeling the feedforward inhibition prevalent in the thalamocortical system [4]. The classical push-pull feedforward model [5] proposed the existence of tuned feed-forward inhibitory neurons with RFs that "mirror" those of the excitatory neurons and supply disynaptic hyper-polarization to the principal cells in layer 4. To account for the discrepancy in the numbers of inhibitory and excitatory cells, it was suggested that these inhibitory neurons are multiplexed (i.e. a single inhibitory neuron supplies inputs to multiple excitatory neurons [6]), though there is uncertainty in the potential implementations of this multiplexing. Assuming that the interneurons mediating the feedforward inhibition is a separate population from the recurrent inhibition population, we show that multiplexing can be implemented naturally when the

representation is overcomplete (Fig. S1). Specifically we rewrite the feedforward transformation ($\Phi^T$) in Eq. (16) as follows:

$$\Phi^T = \Phi_+^T + \Phi_-^T = \Phi_+^T + \Psi\Omega.$$

We can interpret $\Omega$ as the connectivity matrix from the inputs to the feedforward inhibitory interneurons and $\Psi$ as the connectivity matrix from the interneurons to the principal cells. Note that the dimension of $\Omega$ can be arbitrarily assigned as long as the decomposition holds. A simple choice is to make $\Omega$ a full-rank dictionary matrix ($N \times N$) with Gabor-like elements. As a result when the principal cell representation is overcomplete ($\Psi : M \times N$ with $M > N$), fewer feedforward interneurons than principal cells are needed. Furthermore, the RFs of the feedforward interneurons are Gabor-like, similar to those in the classical push-pull model [6]. Note that $\Psi$ is uniquely defined given $\Phi$ and $\Omega$, because in this case $\Omega^{-1}$ exists .

# Global inhibition

While many implementations of inhibitory interneurons are discussed in the main text, an alternative strategy to improve the E/I ratio is to use a global inhibition approach. In the network of Eq. (4), we can offset the baseline excitatory synaptic weights by a constant $c > 1$ to make all of the individual synaptic weights positive: $(c - \langle\phi_j, \phi_i\rangle) > 0, \forall j$. With this offset to the individual weights, the equivalent dynamics model becomes:

$$\tau \dot{u}_i(t) = \langle\phi_i, \mathbf{s}\rangle + \underbrace{\sum_{j\neq m}(c - \langle\phi_j, \phi_i\rangle)a_j(t)}_{\text{Excitatory}} - \underbrace{c\sum_{j\neq m} a_j(t)}_{\text{Global Inhibitory}} - u_i(t) \qquad \text{(S8)}$$

where we now have a single non-orientation tuned inhibitory cell (Fig. S2) that sums over the activity of all principal cells. In biological reality global inhibition is unlikely to manifest in this manner, if at all. A more likely scenario is that this type of interneuron inhibits only a local population of principal cells. This may in fact contribute to diverse orientation tunings of the inhibitory neurons, depending on the local distribution of principal cell orientation tuning properties (e.g. location on the pinwheel).

We note several additional features of this global inhibition implementation of sparse coding. First, the global inhibition rises and falls with the overall excitatory activity in the network. This implies that the model inhibitory neurons have inherently higher firing rates, reminiscent of one class of inhibitory neurons in the cortex that are fast-spiking [7]. Second, the network equation indicates that excitatory cells only connect to those sharing overlapping RFs, reminiscent of the connectivity pattern seen in layer 2/3 where cells with overlapping inputs share connections [8,9], while inhibitory interneurons form dense connections with all neighboring excitatory neurons, similar to the pattern seen in physiology [10]. Third, the excitatory synaptic weights may result from combined Hebbian and anti-Hebbian learning with rates that depend on the overlap of the RFs. Functionally, the connection pattern in Eq. (S8) can be interpreted as a way for excitatory connections to share responsibilities for representing inhibition by shifting the baseline activity to a non-zero value. The cost of such an implementation is that the excitatory population has a higher gain, which may potentially induce a higher sensitivity to noise.

## References


1. Olshausen B, Field D (1996) Emergence of simple-cell receptive field properties by learning a sparse code for natural images. Nature 381: 607–609.

2. Rozell CJ, Johnson DH, Baraniuk RG, Olshausen BA (2008) Sparse coding via thresholding and local competition in neural circuits. Neural Computation 20: 2526–2563.

3. Hyvärinen A, Hurri J, Hoyer P (2009) Natural Image Statistics: A probabilistic approach to early computational vision. Springer-Verlag New York Inc.

4. Isaacson J, Scanziani M (2011) How inhibition shapes cortical activity. Neuron 72: 231–243.

5. Ferster D, Miller K (2000) Neural mechanisms of orientation selectivity in the visual cortex. Annual Review of Neuroscience 23: 441–471.

6. Hirsch J, Martinez L (2006) Circuits that build visual cortical receptive fields. Trends in neurosciences 29: 30–39.

7. Huberman AD, Niell CM (2011) What can mice tell us about how vision works? Trends in Neurosciences 34: 464–473.

8. Yoshimura Y, Dantzker JLM, Callaway EM (2005) Excitatory cortical neurons form fine-scale functional networks. Nature 433: 868–873.

9. Ko H, Hofer SB, Pichler B, Buchanan KA, Sjöström PJ, et al. (2011) Functional specificity of local synaptic connections in neocortical networks. Nature 473: 87–91.

10. Bock DD, Lee WCA, Kerlin AM, Andermann ML, Hood G, et al. (2011) Network anatomy and in vivo physiology of visual cortical neurons. Nature 471: 177–182.
