# Supplementary figures and images for: Modeling Inhibitory Interneurons in Efficient Sensory Coding Models
Source: PLoS Comput Biol. 2015 Jul 14;11(7):e1004353. doi: 10.1371/journal.pcbi.1004353 (PMC4501572; doi:10.1371/journal.pcbi.1004353)

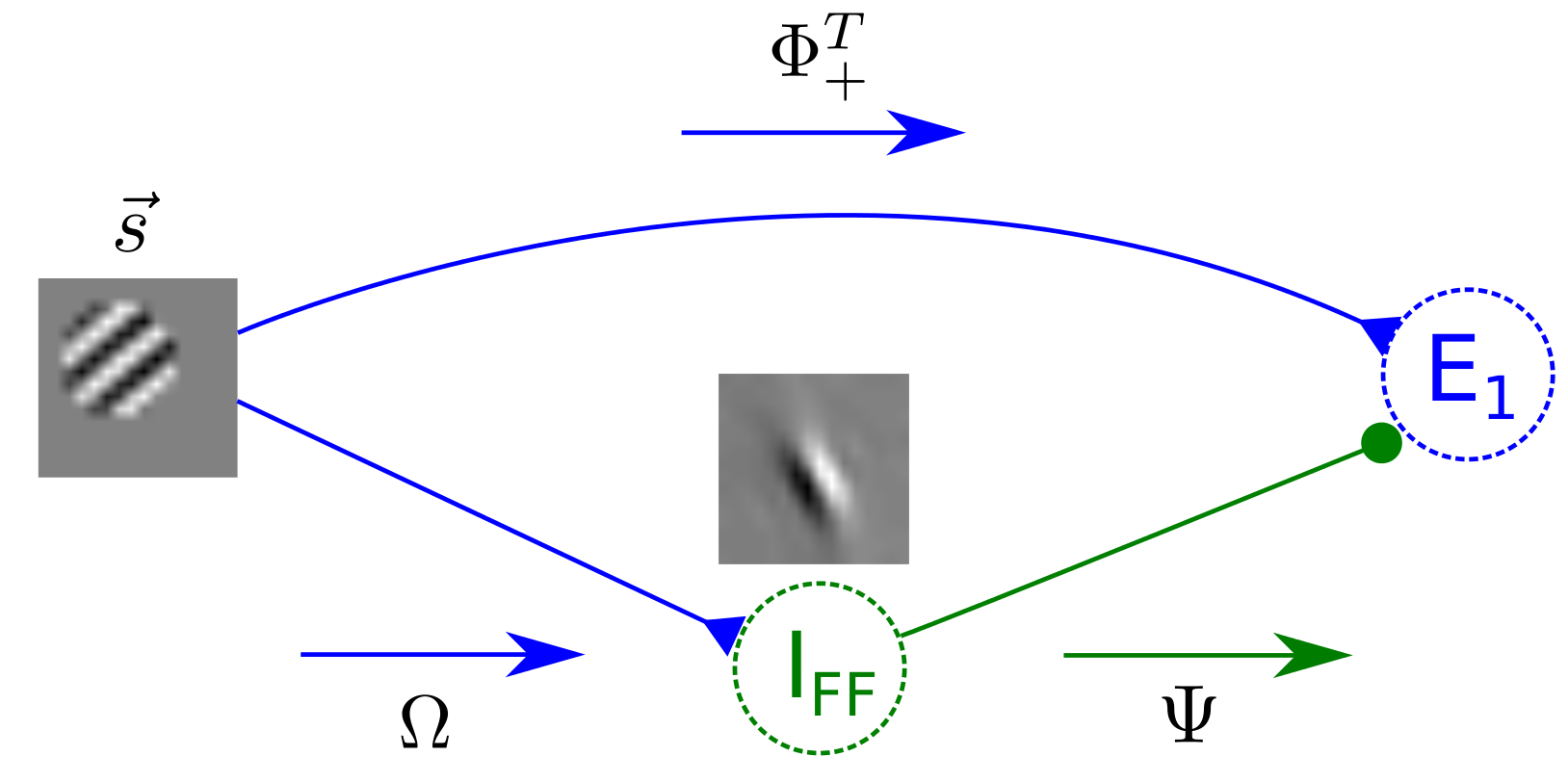

Supplement: S1 Fig — Feedforward push-pull could also be implemented with fewer inhibitory neurons than excitatory neurons. (TIF) [file pcbi.1004353.s002.tif]

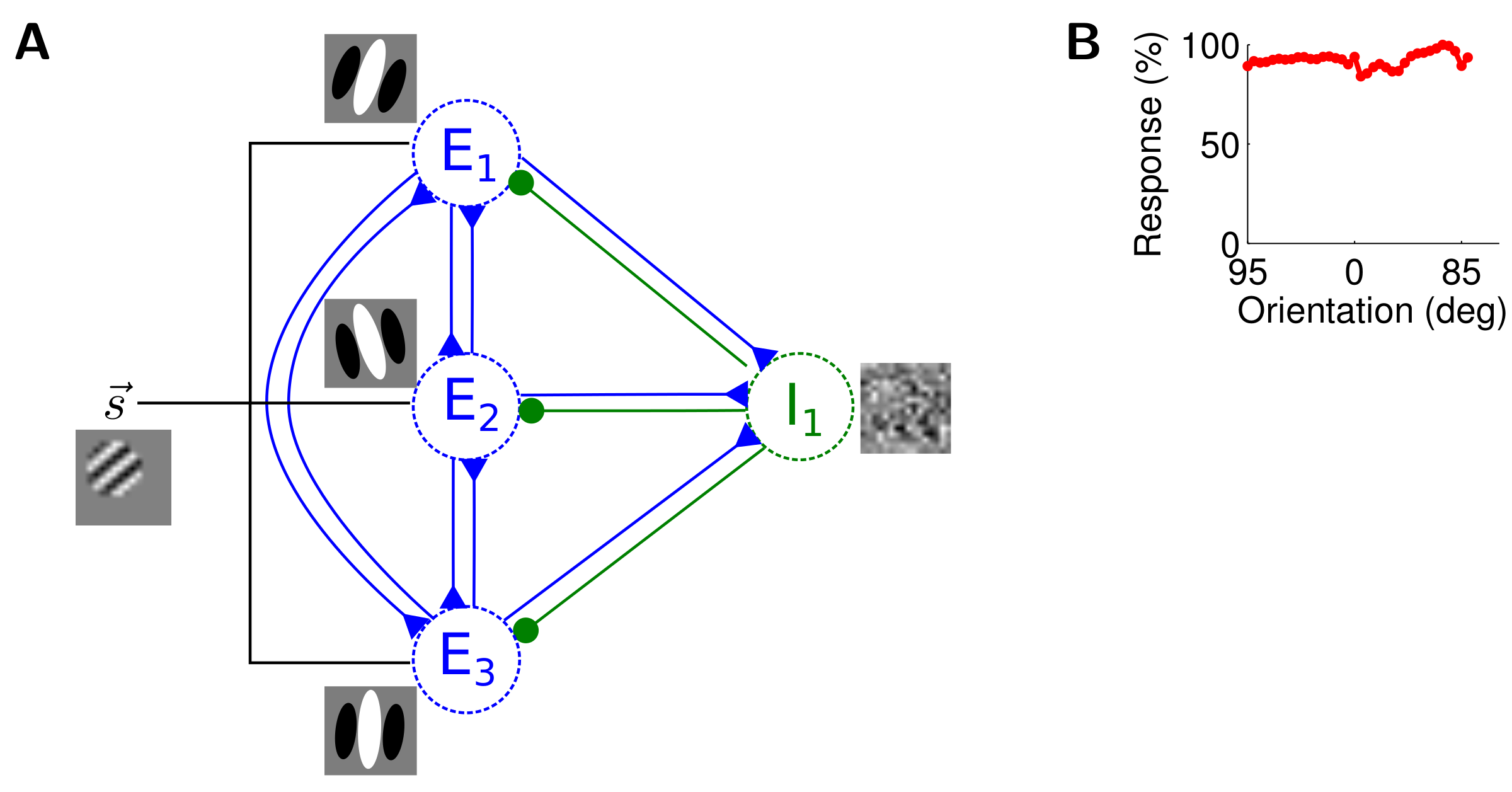

Supplement: S2 Fig — (A) The recurrent network that implements the global inhibition (Eq. (S8)). I1 pools all activities from the excitatory population, weighs them by c, and projects back to the excitatory population. (B) The orientation tuning curve of the inhibitory neuron I1. (TIF) [file pcbi.1004353.s003.tif]
